# Determinants of the Association Between Maternal Anemia and Neonatal Hemoglobin

**DOI:** 10.3390/nu17142292

**Published:** 2025-07-11

**Authors:** Rebecca K. Campbell, Nicole K. Tanna, Julie Hartwig, Catalin S. Buhimschi, Irina A. Buhimschi

**Affiliations:** 1 Division of Epidemiology and Biostatistics, School of Public Health, University of Illinois Chicago, Chicago, IL 60612, USA; 2 College of Medicine, University of Illinois Chicago, Chicago, IL 60612, USA; ntanna2@uic.edu; 3 Department of Obstetrics and Gynecology, College of Medicine, University of Illinois Chicago, Chicago, IL 60612, USA; julie8@uic.edu (J.H.); csb01@uic.edu (C.S.B.); irina@uic.edu (I.A.B.)

**Keywords:** iron deficiency, anemia, nutrition, pregnancy, sex differences, placenta, developmental origins of health and disease

## Abstract

**Background/Objectives:** Iron stores accrued in utero are critical for fetal and infant neurodevelopment. Low neonatal hemoglobin (Hb) may indicate inadequate iron capture and storage. Prior studies differ on whether and under what conditions maternal anemia predicts neonatal Hb; whether sex differences are present is unknown. **Methods:** Maternal and neonatal Hb and sociodemographic and health characteristics were abstracted from electronic medical records for biorepository participants at a tertiary academic medical center. Maternal anemia was defined as Hb < 11 g/dL in trimesters T1 and T3 and Hb < 10.5 g/dL in T2. Adjusted linear regression models were used to estimate associations of maternal anemia with neonatal Hb. Sex differences were evaluated with product terms and stratification. **Results:** In 228 participants with maternal Hb measured, the prevalence of prenatal (pre-delivery) and delivery anemia was 54% and 44%, respectively. Maternal race and ethnicity but no other sociodemographic characteristics were associated with maternal anemia. Neonatal hematology was available for 114 newborns < 7 days old (50%; 52% male). The median (IQR) neonatal Hb was 16.7 g/dL (14.9, 18.0) and did not differ by sex, but it was lower among infants of mothers with vs. without delivery anemia (15.9 vs. 17.1, *p* = 0.032) and those identifying as Black vs. Hispanic or other (16.0, 17.9, 17.0, respectively; *p* = 0.003). Independent associations of maternal anemia and race and ethnicity with neonatal Hb were stronger in males and attenuated to null in females. **Conclusions:** Maternal anemia was highly prevalent and associated sex-specifically with neonatal Hb independent of maternal race and ethnicity. Future studies to replicate these findings with a more comprehensive panel of iron biomarkers are needed. Functional consequences of greater susceptibility to risk factors for low neonatal Hb in male infants need to be further investigated.

## 1. Background

Iron is essential for physiologic processes, including oxygen transport, cellular respiration, and DNA synthesis [1]. Iron transferred across the placenta for fetal use and storage supports growth and development in utero and through the first year of postnatal life [2,3,4]. Inadequate fetal iron accrual is associated with irreversible deficits in neurodevelopment and in heart, lung, and immune system development [3,5,6,7,8,9,10,11]. While the consequences of infant iron deficiency are well described, the extent to which common maternal and pregnancy conditions, including maternal iron deficiency and anemia, put neonates at risk of iron deficiency and developmental deficits are not well understood.

Anemia, defined by low hemoglobin (Hb) relative to age and sex-specific cutoffs, is a readily accessible indicator of advanced iron deficiency, indicating inadequate iron availability for erythropoiesis [12]. In the US, an estimated 8% of women of reproductive age and 3.3% of toddlers have anemia [13], of which at least 50% of cases are thought to be attributable to iron deficiency [14]. National estimates of the prevalence of anemia in pregnant women are 5.3%, 12.7%, and 27.5% in the first, second and third trimesters, respectively [15]. Disparities by race and ethnicity are well described, with rates 2–6 times higher among Hispanic and non-Hispanic Black women compared to non-Hispanic White women [13,15,16]. Anemia rates and disparities have increased over the past two decades [16,17]. In pregnancy, anemia arises secondary to normal hemodilution and reaches a nadir in the second trimester. When iron is sufficiently available, Hb rebounds partially during the third trimester, even as maternal iron stores are depleted to meet fetal iron demands, which peak late in the third trimester [18]. While a recent statement from the United States Preventive Services Taskforce (USPSTF) concluded that neither screening for ID nor iron supplementation in pregnancy is supported by a strong base of evidence [19], many clinical guidelines still call for universal prenatal iron supplementation to meet the iron requirements of pregnancy [20,21].

The extent to which neonatal iron status depends on maternal iron status is debated [5,21,22]. Evidence from direct studies of maternal and infant status is limited because blood draws in healthy infants are often infeasible [22]. A constellation of indirect evidence supports the idea that fetal iron accrual is driven by fetal demand irrespective of maternal iron status except when maternal iron deficiency is severe: cord blood ferritin concentration is much higher than in maternal circulation, iron is actively transferred across a positive concentration gradient from maternal to the fetal circulation, and newborn iron endowment is positively associated with birthweight [5]. However, this argument is predicated on the assumption that neonatal iron deficiency is rare despite widespread maternal iron deficiency. Accumulating evidence suggests this may not be correct. Several small studies suggest that iron deficiency affects around 20% of healthy neonates [23,24,25], much more than previously thought. Additionally, relatively common pregnancy conditions, such as gestational diabetes, obesity, and even psychosocial stress, have been linked to lower neonatal iron status and neurocognitive deficits [26,27,28,29,30]. Finally, multiple recent studies in well-nourished populations found nearly universal iron deficiency in late pregnancy [31,32], suggesting sufficient fetal iron endowment may be tenuous in more pregnancies than was previously appreciated. Taken together, these observations suggest that fetal iron endowment may be more sensitive to maternal conditions, and neonatal iron deficiency is more common than previously appreciated.

The placenta has a critical role in fetal iron accrual, transporting the iron required by the fetus from maternal circulation and storing excess iron to prevent fetal overload [12,33]. Recent studies have shown that the placenta responds to low maternal iron availability by increasing transferrin receptors to acquire more iron from the maternal circulation [34,35]. Evidence is mixed, however, on whether placental ferroportin, which exports iron to the fetal circulation, is responsive to maternal iron deficiency and whether it responds by increasing or decreasing iron export [33,34,36]. The placenta is a sexually dimorphic organ [37,38,39,40]. Sex-specific characteristics include differences in efficiency and nutrient transfer described as more conservative in female vs. male placentas, such that female fetuses are more protected from late pregnancy insults [41,42]. While sex differences in placental iron transporters have not, to our knowledge, been described in the literature, prior studies have observed that male neonates tend to have lower iron biomarkers, and male infants are more susceptible to neurodevelopmental consequences of prenatal exposures that constrain iron accrual, such as gestational diabetes [5,27,43]. More studies are needed to examine sex differences in the relationship between maternal and pregnancy conditions, including prenatal iron deficiency and neonatal iron status. In this study, data from a hospital-based biorepository are used to investigate determinants of maternal and neonatal Hb, including infant sex and determinants of the relationship between maternal and neonatal Hb. We aimed to determine predictors of maternal and neonatal Hb, the relationship between them, and differences by sex.

## 2. Methods

Pregnant people who received prenatal care at The University of Illinois at Chicago (UIC) Hospital & Health Sciences System (UI Health) in Chicago, IL, between October 2020 and June 2023 were enrolled prospectively in a repository of biological specimens and clinical data. The goal of the University of Illinois Chicago Perinatal Research Biorepository is to develop a high-quality and consistent repository of biological specimens and data that advances the understanding of the causes and mechanisms leading to major obstetrical syndromes, including preeclampsia, spontaneous preterm labor, and fetal growth restriction. While the demographic characteristics of the enrolled patients are representative of the delivery population at our academic center, the biorepository is enriched in patients at risk for pregnancy complications. Patients with normal pregnancies and no risk factors are also enrolled, contingent on the availability of the research staff. Patients were eligible to participate in the biorepository if they were pregnant or recently postpartum (within 8 weeks after a viable or non-viable delivery), at least 18 years old, and able to speak and read/write in English or Spanish. Exclusion criteria were known HIV or hepatitis B or C infection and cognitive impairment. For this analysis, twin pregnancies and neonates delivered before 26 weeks gestational age (GA) were excluded. The biorepository protocol was approved by the University of Illinois Chicago Office for the Protection of Human Subjects [Study# 2019-0726]. All participants provided written informed consent.

Pregnancy and delivery details and sociodemographic characteristics were accessed through the Electronic Health Record (EHR) system. Hemoglobin (Hb) values from clinical complete blood counts (CBCs) at the study enrollment visit, at delivery (during the delivery hospital admission, prior to delivery), and the lowest prenatal (pre-delivery) values, along with the total number of prenatal CBCs, were abstracted from the maternal EHR. Linked maternal–infant medical record numbers enabled linking infant birth data, including from a NICU stay if applicable, to maternal prenatal and delivery records. First, neonatal Hb within 7 days after birth was abstracted from the infant EHR. Maternal prenatal and delivery anemia were defined as Hb < 11 g/dL in trimesters 1 and 3 and <10.5 g/dL in trimester 2 [44]. Neonatal anemia was defined as Hb < 13.5 g/dL [45]. At our institution, delaying clamping of the umbilical cord for 30 s after delivery of the baby by vaginal or Cesarean birth is routinely performed, which is known to increase neonatal iron stores [46,47]. Covariates from the EHR were maternal age at enrollment, maternal race and ethnicity, GA at birth, mode of delivery, and infant sex. Pregnancy and delivery complications were abstracted from EHR notes and discharge summaries by a medical student. For this analysis, any hypertensive disorder of pregnancy (HDP) but not chronic hypertension was coded as a dichotomous variable (yes/no) and included as a covariate. No other single complication affected enough pregnancies to be included in the analysis.

Maternal and infant characteristics predictive of the number of maternal or neonatal CBCs were examined with χ^2^ tests. Then, differences in lowest Hb and Hb at delivery by maternal characteristics were evaluated as continuous variables with one-way ANOVA, and differences in the prevalence of anemia at each time point by the same maternal characteristics were quantified with log-binomial regression models. Next, predictors of neonatal continuous Hb and dichotomous anemia were examined with one-way ANOVA and χ^2^ tests, overall and separately for male and female infants.

Finally, the relationships between maternal and neonatal continuous Hb and dichotomous anemia were examined. Scatterplots, correlations, and non-parametric rank sum tests were calculated for all mother–infant pairs and for subsets of the pairs stratified by maternal and infant characteristics to examine linear and non-linear trends. Linear regression models were used to quantify the association between maternal anemia during pregnancy and at delivery and neonatal Hb. Models were repeatedly stratified by infant sex and other maternal covariates. Product terms for the interaction between maternal anemia and each covariate were introduced in separate linear regression models to assess for effect measure modification. All analyses were conducted in Stata 18.

## 3. Results

In 240 eligible pregnancies, the median maternal age was 30 years (Table 1). Nearly 70% of women identified as Black and 22% as Hispanic. Hypertensive disorders of pregnancy were common, affecting 41% of the pregnancies. Just over half were delivered by C-section, and the median gestational age at delivery was 37 weeks 5 days. Of the enrolled pregnancies, 225 (94.5%) had at least one CBC. The median lowest prenatal Hb was 10.7, and 54% had anemia according to their lowest prenatal Hb. At delivery, the median Hb was 11.15, and 44% had anemia.

Maternal race and ethnicity were associated with prenatal and delivery Hb (F-test *p*-value = 0.004 and 0.015, respectively) (Table 2). The risk of prenatal anemia was 43% lower in Hispanic vs. Black pregnant people, while the risk of delivery anemia did not differ significantly by race and ethnicity (Table 2). The number of prenatal CBCs had a positive dose–response association with the risk of prenatal anemia, but for delivery anemia, only having no prenatal CBCs was associated with an elevated risk of anemia at delivery. Delivery but not prenatal Hb was higher in those with HDP (11.25 vs. 11.0, *p* = 0.028) and those delivered by C-section (11.3 vs. 11.0, *p* = 0.050). Maternal Hb and anemia were not associated with fetal sex or with gestational age at delivery.

Neonatal CBCs were available for slightly less than half of the enrolled pregnancies (n = 114). The percent with neonatal CBCs differed by HDP, mode of delivery, and gestational age at birth, with those with a maternal HDP, C-section delivery, and preterm birth more likely to have a neonatal Hb measured (Table 3). Race and ethnicity and infant sex were not associated with Hb missingness.

**Figure 1:**
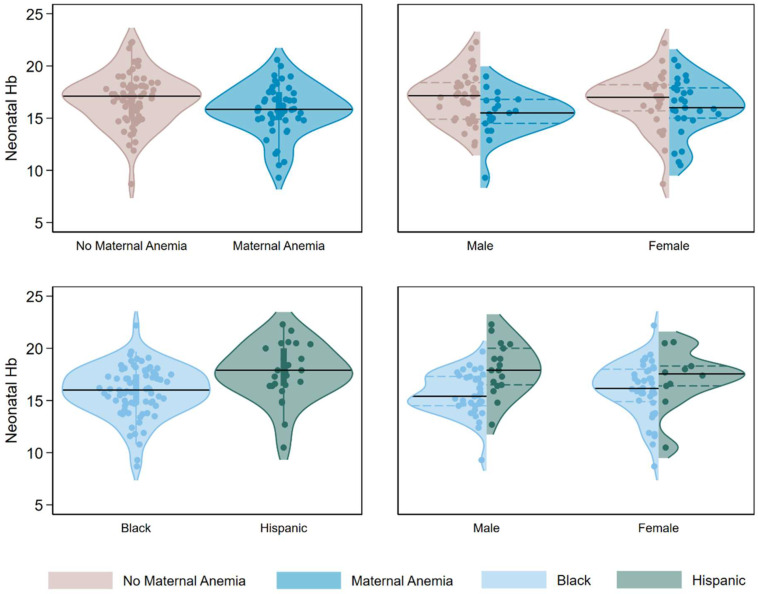
Overall and sex-specific associations of maternal anemia at delivery and race and ethnicity with neonatal Hb, UI Health Perinatal Biorepository, 2020–2023. Maternal anemia at delivery and Black (vs. Hispanic) race or ethnicity are associated with lower neonatal Hb. When stratified by biological sex, the association of maternal anemia and neonatal Hb is stronger among male infants (β = −1.58, 95% CI = −2.87, −0.30, *p*-value = 0.017) and attenuated to near the null in female infants (β = −0.55, 95% CI = −2.04, 0.94, *p*-value = 0.461). Similarly, when stratified by biological sex, the difference in neonatal Hb between infants of Black and Hispanic mothers is larger among male infants (β = −2.47, 95% CI = −3.74, −1.19, *p*-value < 0.001) and attenuated to near the null in female infants (β = −1.0, 95% CI = −2.92, 0.89, *p*-value = 0.289).

The median neonatal Hb was 16.7 g/dL (IQR: 14.9, 18.0) (Table 3). Neonatal Hb was the highest in infants of Hispanic mothers and lowest in infants of Black mothers (17.8 g/dL vs. 16.0 g/dL, F-test *p*-value = 0.0188). Maternal anemia at delivery was associated with significantly lower neonatal Hb (15.9 vs. 17.1, *p* = 0.0417), while neonatal Hb did not differ by pre-delivery anemia, HDP, mode of delivery, or gestational age at birth. When stratified by infant sex, the associations of maternal race and ethnicity and delivery anemia with neonatal Hb persisted and were more pronounced in male infants, whereas associations were weaker and not statistically significant in female infants (Figure 1). As very few infants had Hb values below the established cutoff for anemia, only analyses using continuous neonatal Hb are presented.

On average, neonates of mothers with anemia at delivery had Hb that was lower by 0.99 g/dL (95% CI: −1.94, −0.04) vs. neonates of mothers without delivery anemia. This association was stronger in males and in infants of Black mothers, while no association between maternal anemia and infant Hb was observed for Hispanic mothers (ns were too small to analyze this in White, Asian, or “other” mothers). Effect measure modification by sex, race and ethnicity, and pregnancy characteristics (HDP, GA) did not reach statistical significance. In multivariable models, delivery anemia and maternal race and ethnicity were independently associated with neonatal Hb in male neonates and in males and females combined (Table 4).

## 4. Discussion

In a hospital-based biorepository of predominantly non-Hispanic Black and Hispanic women, the prevalence of anemia during pregnancy and at delivery was high, differed by race and ethnicity, and predicted neonatal Hb. The relationships of maternal anemia and race and ethnicity with neonatal Hb differed by infant sex, with stronger inverse associations in males compared to females.

More than half of the participants had anemia during pregnancy, and 45% had anemia at delivery. These prevalence estimates are higher than those recently reported in California and Ontario, Canada [16,48]. Differences may stem from different underlying population risks or from a selection of higher-risk pregnancies in the sample for the present study. Rates of both prenatal and delivery anemia differed by race and ethnicity, with the highest rates among non-Hispanic Black women. Racial and ethnic differences in anemia prevalence are widely reported, with non-Hispanic Black women at greatest risk, as we saw in this study [13,16,49]. The risk among Hispanic women is less clear, however, with some studies suggesting moderately elevated risk of anemia among Hispanic women compared to White women and others showing no increased risk [13,15,16]. In the present study, Hispanic women had a lower prevalence of anemia than White women, but the sample of White women was very small. Prior studies show heterogeneity in the risk of anemia within Hispanic women by country or region of family origin and U.S. region of residence [50], which could contribute to discrepant findings in the literature.

Delivery Hb but not prenatal Hb was higher among those with HDP and those delivered by C-section. It is possible that in some cases, delivery anemia status reflects inflammation associated with labor or other complications of pregnancy or delivery, for example, hemoconcentration associated with preeclampsia. Adjustments for pregnancy complications and mode of delivery may be necessary to accurately assess the relationship between delivery Hb or anemia and adverse outcomes but would require a large sample with greater variation in comorbidity diagnoses. In a larger sample, differences in maternal anemia by gestational age at delivery or infant sex may also be observable.

In this study, neonatal Hb was associated with maternal race and ethnicity and maternal delivery anemia but no other maternal or pregnancy characteristics. As infant iron stores are understood to increase with increasing gestational age, our finding of no relationship between Hb and gestational age at birth is likely due to the relatively small variability in gestational ages present in the sample with neonatal Hb available since infants born near or at term were much less likely to have a Hb measurement in their medical chart. There were also no observed differences in neonatal Hb by infant sex or mode of delivery [46,47]. The observation that delivery but not prenatal anemia is associated with lower neonatal Hb could be due to several factors. Mid-pregnancy anemia may reflect normal hemodilution that resolves later in pregnancy and does not harm the placenta or fetus, whereas delivery anemia reflects true depletion of iron stores that may constrain the iron available to the fetus. Additionally, anemia detected in mid-pregnancy should prompt iron treatment, so anemia at delivery could reflect iron deficiency that is refractory to supplementation, non-iron deficiency anemia, or low adherence to recommended treatment. Data on prenatal iron supplementation dosing and adherence and iron status biomarkers would help elucidate these issues but were not available in this EHR dataset.

In models stratified by infant sex, the relationship between maternal delivery anemia and neonatal Hb was stronger in male infants and much less pronounced in female infants. Similarly, for maternal race and ethnicity, in stratified models, neonates of non-Hispanic Black mothers with anemia at delivery had lower Hb relative to non-Hispanic Black mothers without delivery anemia, while neonates of Hispanic mothers with and without anemia did not differ in their Hb value. In multivariable models, delivery anemia and race and ethnicity were independently associated with neonatal Hb. These associations persisted in stratified models among boys but not girls. These observations suggest that infant sex and maternal race and ethnicity may each modify the risk for neonatal iron endowment associated with maternal anemia. In the present study, these subgroup differences (i.e., effect modification) did not reach statistical significance. More research is needed in a sample similarly enriched for racial and ethnic diversity and with a larger sample of neonates having Hb measured. To our knowledge, this is the first study to suggest differential fetal susceptibility to maternal iron deficiency by fetal sex and by maternal race and ethnicity. These differences may be important, both for interpreting discrepancies in the prior literature and for considering targeted interventions to protect fetal and neonatal iron status. More research is needed to replicate these findings and incorporate direct measures of maternal and infant iron status to advance the understanding and practice in this area.

The present study has several strengths, including access to linked maternal and neonatal medical record data with a high rate of neonatal Hb measured. Additionally, the sample was made up predominantly of Black and Hispanic women, who are two of the highest-risk groups for anemia. Our present exploration of sex differences is novel in the literature on this topic. In addition to allowing us to generate more precise estimates, observed sex differences offer a new lens through which to interpret fetal susceptibility to maternal low iron. At the same time, this study has some important limitations. This was a relatively small preliminary exploration of data collected for clinical but not research purposes. However, using EHR data enabled us to include a population that is historically underrepresented in biomedical research and to access neonatal hematologic markers, which are not readily accessible outside of NICU care for research purposes. Participants were not randomly sampled from UI Health pregnant patients, and, in fact, preference was given to enroll high-risk or complicated pregnancies into the biorepository, given its primary aims of studying preeclampsia, spontaneous preterm labor, and fetal growth restriction. Additionally, the mechanisms leading to the selection of infants into the study were non-random in that, of the enrolled pregnancies, only neonates admitted to the NICU had hematology measured in the days after birth as part of the standard of care. Given the relative lack of prior literature on this topic, it is not clear what effect, if any, these selection mechanisms would have on the findings, but they may limit the generalizability of the results to the general population. Further research is needed to replicate and expand on these findings in a more representative cohort. Future studies that can include biomarkers of iron status and inflammation in addition to Hb would also help elucidate the dynamics of maternal–fetal iron transfer when maternal iron is low. Finally, the relatively small sample limited the complexity of analyses that could be conducted, and some desired variables, such as maternal antenatal iron supplementation and timing of umbilical cord clamping in individual deliveries, were not available for analysis.

To follow up on these findings, a larger study with more in-depth iron status assessments, preferably at multiple prenatal time points in the mothers, is needed to quantify sex differences in fetal exposure to maternal anemia and iron deficiency. Further exploration of comorbidities and their timing relative to iron deficiency and anemia is also needed. If sex differences in fetal susceptibility to maternal anemia are reproduced, this may have implications for monitoring and managing anemia and iron deficiency in pregnancy. It suggests that proactive management of prenatal ID may be especially important for pregnancies carrying male fetuses for infants and possibly also maternal health outcomes.

## 5. Conclusions

In the present study of predominantly Black and Hispanic pregnant people and their neonates at an academic medical center in Chicago, maternal anemia and Black race were associated with lower neonatal Hb, and both associations were stronger in male vs. female infants. Male infants may be especially susceptible to maternal iron deficiency and other conditions that constrain iron accrual. More studies are needed to determine the need and suitable approach for tailoring the management of pregnancy anemia by fetal sex and other risk factors.

## Figures and Tables

**Table 1:** Maternal, pregnancy, and neonate characteristics of singleton pregnancies in the UI Health Perinatal Biorepository, 2020–2023.

Characteristic	n	n (%) or Median (IQR)
Maternal and Pregnancy		
Age (years), median (IQR)	238	30 (26, 35)
Race and ethnicity	235	
Asian		5 (2.1)
Black		163 (69.4)
Hispanic		52 (22.1)
Other		1 (0.4)
White		14 (6.0)
Any HDP	238	
No		140 (58.8)
Yes		98 (41.2)
Mode of delivery	238	
Cesarean		114 (47.9)
Vaginal		124 (52.1)
GA at delivery (days), median (IQR)	237	264 (249, 273)
Number of CBCs	236	
Zero		12 (5.1)
One		28 (11.9)
Two		57 (24.2)
Three or more		139 (58.9)
Lowest prenatal Hb (g/dL), median (IQR)	225	10.7 (10, 11.6)
Prenatal anemia	223	
No		102 (45.7)
Yes		121 (54.3)
Delivery Hb (g/dL), median (IQR)	230	11.1 (10.4, 12.1)
Delivery anemia	230	
No		127 (55.2)
Yes		103 (44.8)
Neonate		
Sex	228	
Male		119 (52.2)
Female		109 (47.8)
Hb (g/dL), median (IQR)	113	16.7 (14.9, 18)

**Table 2:** Associations of maternal and pregnancy characteristics with hemoglobin (Hb) and anemia during pregnancy and at delivery, UI Health Perinatal Biorepository, 2020–2023.

	Lowest Prenatal Hb		Prenatal Anemia		Delivery Hb		Delivery Anemia	
	n	Median (IQR)	n (%)	RR (95% CI)	n	Median (IQR)	n (%)	RR (95% CI)
Age, years								
18–24	41	10.5 (10, 11.1)	27 (65.9)	1.00 (Reference)	42	11 (10.2, 11.9)	21 (50)	1.00 (Reference)
25–34	117	10.9 (10.1, 11.6)	57 (49.6)	0.75 (0.56, 1.00)	120	11.1 (10.4, 11.9)	53 (44.2)	0.88 (0.61, 1.27)
35+	67	10.6 (9.6, 11.7)	37 (55.2)	0.84 (0.62, 1.14)	68	11.3 (10.6, 12.2)	29 (42.7)	0.85 (0.57, 1.28)
*p*-Value		0.567		0.153		0.736		0.724
Race and ethnicity								
Black	152	10.6 (9.9, 11.2)	91 (60.3)	1.71 (1.15, 2.53)	157	11 (10.3, 11.8)	78 (49.7)	1.41 (0.94, 2.11)
Hispanic	51	11.5 (10.4, 12.1)	18 (35.3)	1.00 (Reference)	51	11.6 (10.7, 12.5)	18 (35.3)	1.00 (Reference)
Other	18	10.7 (10.1, 12.1)	9 (52.9)	1.5 (0.84, 2.68)	18	11.6 (10.9, 12.5)	5 (27.8)	0.79 (0.34, 1.81)
*p*-Value		0.003		0.027		0.006		0.0989
Any HDP								
No	131	10.7 (10.0, 11.7)	68 (52.3)	1.00 (Reference)	134	11.0 (10.3, 11.9)	64 (47.8)	1.00 (Reference)
Yes	94	10.7 (9.9, 11.6)	53 (57.0)	1.09 (0.86, 1.39)	96	11.3 (10.5, 12.3)	39 (40.6)	0.85 (0.63, 1.15)
*p*-Value		0.906		0.486		0.025		0.29
Mode of delivery								
Cesarian	104	10.8 (10.1, 11.7)	52 (50.5)	0.88 (0.69, 1.12)	109	11.3 (10.6, 12.3)	42 (38.5)	0.76 (0.57, 1.03)
Vaginal	121	10.6 (9.9, 11.5)	69 (57.5)	1.00 (Reference)	121	10.9 (10.2, 11.9)	61 (50.4)	1.00 (Reference)
*p*-Value		0.832		0.299		0.043		0.075
Infant sex								
Male	114	10.7 (10.1, 11.6)	61 (53.5)	1.00 (Reference)	117	11.2 (10.6, 12.2)	46 (39.3)	1.00 (Reference)
Female	102	10.7 (9.9, 11.7)	55 (54.5)	1.02 (0.79, 1.30)	106	11.0 (10.4, 12)	51 (48.1)	1.22 (0.91, 1.65)
*p*-Value		0.404		0.889		0.098		0.232
GA at delivery								
Early preterm	30	10.5 (9.7, 11.7)	17 (56.7)	1.08 (0.74, 1.6)	32	11.4 (10.1, 12.9)	14 (43.8)	0.91 (0.58, 1.45)
Late preterm	49	11.1 (9.9, 11.9)	23 (46.9)	0.9 (0.62, 1.31)	47	11.3 (10.5, 12.3)	19 (40.4)	0.84 (0.55, 1.29)
Early term	77	10.6 (9.9, 11.5)	46 (60.5)	1.16 (0.87, 1.55)	78	11.1 (10.3, 11.8)	35 (44.9)	0.94 (0.66, 1.32)
Full term	68	10.8 (10.4, 11.5)	35 (52.2)	1.00 (Reference)	73	11 (10.4, 11.7)	35 (48.0)	1.00 (Reference)
*p*-Value		0.633		0.498		0.136		0.883
CBCs								
Zero	0	0	N/A	N/A	12	10.6 (9.5, 11.1)	9 (75.0)	1.69 (0.99, 2.88)
One	27	11.5 (10.4, 12.7)	8 (30.8)	1.00 (Reference)	27	11.4 (10.3, 12.8)	12 (44.4)	1.00 (Reference)
Two	57	11 (10.5, 11.8)	23 (40.4)	1.31 (0.68, 2.53)	55	11.3 (10.7, 12.3)	20 (36.4)	0.82 (0.47, 1.41)
Three or more	139	10.4 (9.6, 11.4)	88 (63.8)	2.07 (1.15, 3.74)	134	11.1 (10.4, 11.9)	60 (44.8)	1.01 (0.63, 1.60)
*p*-Value		0.000		0.003		0.158		0.017

N/A—not applicable.

**Table 3:** Associations of maternal and pregnancy characteristics with neonatal Hb, UI Health Perinatal Biorepository, 2020–2023.

	All			Male		Female	
	Missing, n (%)	Hb, Median (IQR)	*p*-Value ^a^	Hb, Median (IQR)	*p*-Value ^a^	Hb, Median (IQR)	*p*-Value ^a^
Age			0.974		0.722		0.743
18–24 y	24 (57.1)	16.6 (14.9, 17.4)		16.7 (16.2, 17.3)		15.7 (14.8, 18.3)	
25–34 y	70 (55.1)	16.2 (14.9, 17.7)		15.9 (14.7, 17.5)		16.7 (15.7, 18.2)	
35+ y	31 (44.9)	17.0 (15, 18.4)		18.0 (15.1, 18.6)		16.8 (13.8, 18.1)	
Race and ethnicity			0.003		0.001		0.541
Black	88 (54.0)	16.0 (14.7, 17.5)		15.4 (14.5, 17.3)		16.2 (14.9, 18)	
Hispanic	25 (48.1)	17.9 (16.4, 20.0)		17.9 (16.5, 20)		17.6 (16.4, 18.3)	
Other	11 (57.0)	17.0 (15.9, 18.0)		17.3 (16.7, 17.4)		16.7 (15, 18.6)	
HDP			0.484		0.82		0.273
No	89 (62.9) ^b^	16.7 (14.9, 18.1)		16.8 (15.0, 18.0)		16.6 (14.8, 18.2)	
Yes	37 (37.8)	16.7 (15.0, 18.0)		16.5 (14.8, 17.8)		17.1 (15.7, 18.1)	
Prenatal anemia			0.135		0.153		0.487
No	51 (50.0)	17.1 (15.0, 18.2)		17.2 (14.8, 18.1)		17.1 (15.6, 18.3)	
Yes	65 (53.7)	15.9 (14.8, 17.5)		16.2 (14.9, 17.5)		16.1 (15.7, 18.1)	
Delivery anemia			0.032		0.017		0.462
No	63 (49.6)	17.1(14.9, 18.2)		17.2 (14.9, 18.4)		17.0 (15.7, 18.2)	
Yes	57 (55.3)	15.85(14.8, 17.5)		15.5 (14.5, 16.8)		16.0 (15.0, 17.9)	
Mode of delivery			0.3		0.24		0.752
Cesarian	45 (39.5) ^b^	16.4 (14.9, 18)		16.3 (14.9, 18.0)		16.6 (15.0, 18.1)	
Vaginal	80 (64.5)	17.1 (15.2, 18.0)		17.0 (14.8, 17.9)		17.1 (15.7, 18.2)	
Infant sex			0.805				
Male	60 (50.4)	16.7 (14.8, 17.9)					
Female	55 (50.5)	16.7 (15, 18.2)					
GA at birth			0.392		0.246		0.835
Early preterm	4 (11.8) ^b^	16.8 (13.8, 18)		16.5 (13.8, 17.7)		17.1 (15.4, 18.1)	
Late preterm	9 (18.4)	16.7 (15, 18.4)		16.4 (15, 17.3)		17.1 (16.1, 18.8)	
Early term	54 (67.5)	17.4 (15.2, 18.3)		17.9 (15.2, 19.0)		16.9 (14.9, 18.2)	
Full term	57 (77.0)	15.9 (15, 17.1)		16.5 (15.2, 17.4)		15.9 (15, 16.6)	

^a^ F-test *p*-values from ANOVA for continuous Hb by each maternal, pregnancy, or neonate characteristic. ^b^ The percent of missing neonatal Hb differs by the levels of the characteristic at *p* < 0.001.

**Table 4:** Multivariable analysis of characteristics associated with neonatal Hb, overall and stratified by infant sex, UI Health Perinatal Biorepository, 2020–2023.

	n	B (95% CI)	*p*-Value
Overall	107		
Delivery anemia			0.027
No		0.00 (Reference)	
Yes		−1.07 (−2.02, −0.13)	
Race and ethnicity			0.004
Black		−1.84 (−2.93, −0.75)	
Hispanic		0.00 (Reference)	
Other		−0.74 (−2.66, 1.18)	
Infant sex			0.663
Male		0.23 (−0.71, 1.17)	
Female		0.00 (Reference)	
Male infants	55		
Delivery anemia			0.064
No		0.00 (Reference)	
Yes		−1.19 (−2.45, 0.07)	
Race and ethnicity			0.005
Black		−2.26 (−3.59, −0.94)	
Hispanic		0.00 (Reference)	
Other		−1.05 (−3.23, 1.14)	
Female infants	52		
Delivery anemia			0.274
No		0.00 (Reference)	
Yes		−0.83 (−2.34, 0.68)	
Race and ethnicity			0.429
Black		−1.19 (−3.12, 0.73)	
Hispanic		0.00 (Reference)	
Other		−0.27 (−3.82, 3.29)	

## Data Availability

The data presented in this study are available on request from the corresponding author due to privacy restrictions.
